# Novel Genetic Prognostic Signature for Lung Adenocarcinoma Identified by Differences in Gene Expression Profiles of Low- and High-Grade Histological Subtypes

**DOI:** 10.3390/biom12020160

**Published:** 2022-01-19

**Authors:** Chia-Ching Chang, Min-Shu Hsieh, Mong-Wei Lin, Yi-Hsuan Lee, Yi-Jing Hsiao, Kang-Yi Su, Te-Jen Su, Sung-Liang Yu, Jin-Shing Chen

**Affiliations:** 1 Department of Clinical Laboratory Sciences and Medical Biotechnology, College of Medicine, National Taiwan University, Taipei 10048, Taiwan; d05424003@ntu.edu.tw (C.-C.C.); d98455002@ntu.edu.tw (Y.-J.H.); suky@ntu.edu.tw (K.-Y.S.); g9255002@gmail.com (T.-J.S.); 2 Department of Pathology, National Taiwan University Hospital and National Taiwan University College of Medicine, Taipei 100225, Taiwan; mshsieh065@gmail.com (M.-S.H.); yihsuan65@gmail.com (Y.-H.L.); 3 Department of Surgery, National Taiwan University Hospital and National Taiwan University College of Medicine, Taipei 100225, Taiwan; mwlin@ntu.edu.tw; 4 Department of Surgical Oncology, National Taiwan University Cancer Center, Taipei 106037, Taiwan

**Keywords:** histological subtype, lung adenocarcinoma, prognosis, RNA sequencing

## Abstract

The 2021 WHO classification proposed a pattern-based grading system for early-stage invasive non-mucinous lung adenocarcinoma. Lung adenocarcinomas with high-grade patterns have poorer outcomes than those with lepidic-predominant patterns. This study aimed to establish genetic prognostic signatures by comparing differences in gene expression profiles between low- and high-grade adenocarcinomas. Twenty-six (9 low- and 17 high-grade adenocarcinomas) patients with histologically “near-pure” patterns (predominant pattern comprising >70% of tumor areas) were selected retrospectively. Using RNA sequencing, gene expression profiles between the low- and high-grade groups were analyzed, and genes with significantly different expression levels between these two groups were selected for genetic prognostic signatures. In total, 196 significant candidate genes (164 upregulated and 32 upregulated in the high- and low-grade groups, respectively) were identified. After intersection with The Cancer Genome Atlas–Lung Adenocarcinoma prognostic genes, three genes, exonuclease 1 (EXO1), family with sequence similarity 83, member A (FAM83A), and disks large-associated protein 5 (DLGAP5), were identified as prognostic gene signatures. Two independent cohorts were used for validation, and the areas under the time-dependent receiver operating characteristic were 0.784 and 0.703 in the GSE31210 and GSE30219 cohorts, respectively. Our result showed the feasibility and accuracy of this novel three-gene prognostic signature for predicting the clinical outcomes of lung adenocarcinoma.

## 1. Introduction

The International Association for the Study of Lung Cancer, American Thoracic Society, and European Respiratory Society proposed a new classification system for lung adenocarcinoma in 2011 [1]. The new classification system divides lung adenocarcinoma into five subtypes (lepidic, acinar, papillary, micropapillary, and solid), and the effect of the new classification on the prediction of survival rate and recurrence has been reported [2,3]. Patients with micropapillary- and solid-predominant adenocarcinomas have a higher recurrence rate than patients with lepidic-predominant adenocarcinomas. In addition, patients with early-stage lung adenocarcinoma with high-grade subtypes (solid and micropapillary) have higher recurrence rate and poorer prognosis than those with the low-grade subtype (lepidic) after sublobar resection [4,5,6]. Therefore, the histological subtype has been well-documented as an important prognostic factor for early-stage lung adenocarcinoma [2,3,4,5,6].

Although the predominant pattern group can serve as a prognostic factor in overall survival (OS) and recurrence probability, most patients exhibit mixed-type lung adenocarcinomas. Hence, the histological classification of lung adenocarcinomas needs to be improved [7,8,9]. In addition, interobserver disagreement exists in the determination of histological subtypes [7,8,9]. Therefore, revealing the genetic prognostic signature for early-stage lung adenocarcinoma based on histological subtypes may help physicians predict the survival and recurrence of patients more accurately.

This study aimed to explore the relationship between histological subtype and expression profiles individually and to focus on the low-grade subtype (lepidic) and high-grade subtypes (micropapillary and solid).

## 2. Materials and Methods

### 2.1. Patient Populations

The investigations were performed in a retrospective cohort of 26 individuals with surgically resected lung adenocarcinoma who were managed at the National Taiwan University Hospital between 1 January 2011, and 15 November 2021. The inclusion criteria were as follows: patients (1) with lung adenocarcinoma and (2) with a pathologically proven “near-pure” (>70%) single histological subtype of lepidic, solid, or micropapillary lung adenocarcinomas [10]. The Hospital’s Research Ethics Committee approved this study (project approval no. 201610057RINB), and all patients provided written informed consent.

Preoperative staging procedures included chest radiography, blood chemistry analysis and serum carcinoembryonic antigen (CEA) measurement, computed tomography of the chest, abdomen, and brain; bone scanning or positron emission tomography; and pulmonary function tests. All patients underwent standard lung tumor excision and mediastinal lymph node dissection. Clinicopathological parameters, including age, sex, smoking status, preoperative serum CEA level, underlying malignant disease, lung cancer family history, surgical procedure (wedge resection, segmentectomy, or lobectomy), method of surgical approach (thoracotomy or video-assisted thoracoscopic surgery), and clinical outcomes were collected from the chart review.

### 2.2. Histopathological Analysis

All specimens were fixed in formalin and sectioned for microscopic examination with hematoxylin and eosin staining. An experienced thoracic pathologist (M-S Hsieh) performed histopathological studies according to the 2021 World Health Organization (WHO) criteria [11]. Tumor sizes, histological patterns, and pathological features, including tumor cell type, grading, vascular and/or visceral pleural invasions, lymphovascular invasion, spread through airspaces, section margins, and regional lymph node metastasis, were obtained. Tumor spread through air spaces (STAS) was defined as tumor cells within air spaces in the lung parenchyma at a distance of at least one alveolus away from the main tumor [12]. The subtype percentages of each lung tumor, including lepidic, low-grade acinar, papillary, high-grade acinar (including cribriform or complex glandular pattern), micropapillary, and solid types, were also recorded according to the newly proposed grading system of the 2021 WHO [11]. In this study, low-grade adenocarcinomas were defined as having a predominantly lepidic pattern (>70% of total tumor part) with no or less than 20% high-grade pattern, whereas high-grade adenocarcinomas were defined as having either solid or micropapillary as the predominant pattern (>70% of total tumor part). The selected low-grade and high-grade cases were subjected to RNA sequencing.

### 2.3. Total RNA Sequencing Library Construction

A minimum of 1 µg of human total RNA was required for library preparation. Ribosomal RNA was removed using rRNA Removal Mix at 68 °C for 5 min. rRNA removal beads were applied to bind rRNA, and the clear supernatant was transferred to a new 1.5-mL LoBind tube. Elute, Prime, and Fragment High Mix were used to fragment the clear RNA. Double-strand cDNA was synthesized using the First Strand Synthesis Act D Mix and Second Strand Marking Master Mix. After adding A-tailing Mix to the adenylate 3’ ends, the sequencing adaptors were ligated immediately. The cDNA fragments were enriched by 15 cycles of polymerase chain reaction amplification and qualified using Qubit 2.0 Fluorometer (Life Technologies, Carlsbad, CA, USA) and 2200 TapeStation (Agilent Technologies, Santa Clara, CA, USA), respectively.

### 2.4. Differential Gene Expression Analysis

**Figure 1:**
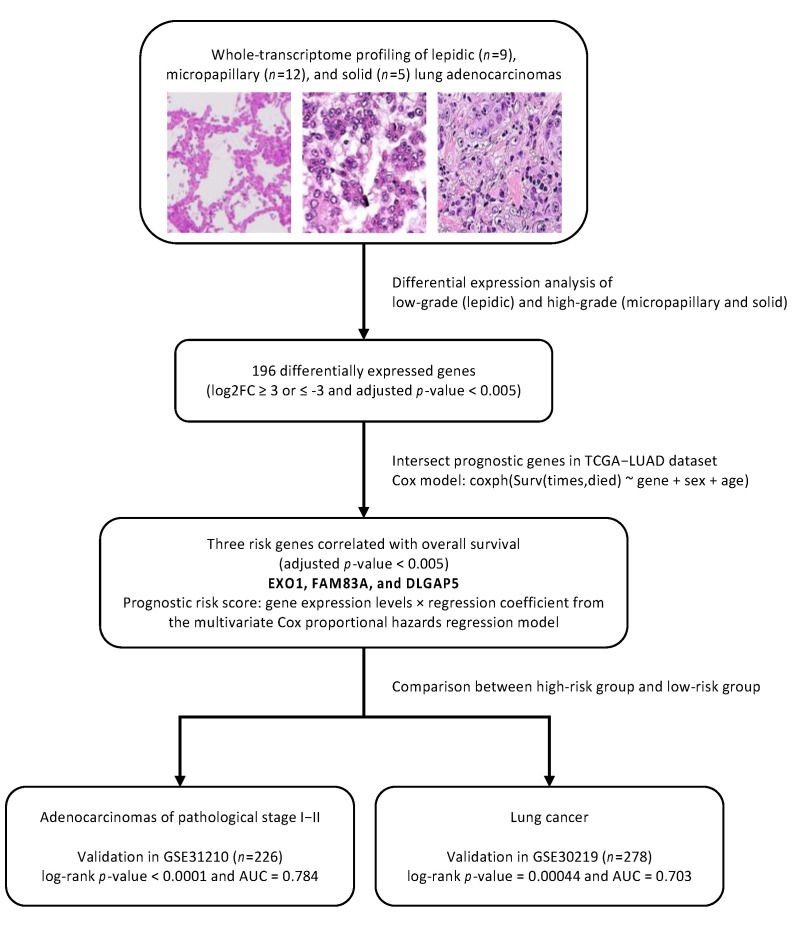
Flowchart of a three-gene prognostic signature construction and validation.

After preparation according to the manufacturer’s protocol, bioinformatic analysis was performed initially from converting and de-multiplexing BCL basecall files to Fastq files using the bcl2fastq tool (Illumina Inc., San Diego, CA, USA) following an Illumina user guide. Adapter sequences and low-quality bases were trimmed using Atropos [13]. The trimmed paired-end reads were mapped to the GRCh37 build of the human genome reference using STAR [14]. Mapped reads in SAM format were transferred to the compressed BAM format by SAMtools [15]. Duplicate reads in the BAM files were marked using the Picard utility [16]. Gene-specific read counting was performed using featureCounts [17] according to the GENCODE gene model [18]. For differential gene expression analysis, DESeq2 [19] was applied to the gene table of the raw read counts. The criteria of significance were the adjusted *p*-value (false discovery rate) being less than 0.005 and the log2 fold change being greater than and equal to 3 or less than and equal to −3 (Figure 1).

### 2.5. Construction and Validation of Prognostic Genes

We intersected the differentially expressed genes to the significant prognostic genes from The Cancer Genome Atlas-Lung Adenocarcinoma (TCGA-LUAD) obtained from OncoLnc with an adjusted *p*-value less than 0.005 [20,21]. A prognostic risk score was calculated based on the expression of these three genes multiplied by their Cox coefficients. Two validation cohorts, 226 patients with adenocarcinomas of pathological stage I–II (GSE31210) [22] and 278 patients with adenocarcinomas and squamous carcinomas (GSE30219) [23], were divided into the high-risk and low-risk groups based on the median of the prognostic risk score. To examine the prognostic performance, a time-dependent receiver operating characteristic (ROC) curve was generated for the areas under the ROC curve.

## 3. Results

### 3.1. Patient Clinicopathological Characteristics

Of the 26 patients included in the study cohort, 9 (34.6%) and 17 (65.4%) had low-grade subtype (lepidic) and high-grade subtype (solid or micropapillary) lung adenocarcinomas, respectively (Table 1). All tumors were pathologically proven to have a “near-pure” (>70%) single histological subtype. The mean follow-up period was 44.1 months (range, 9–117 months). Most patients were nonsmokers (73.1%). Females accounted for 53.8% of the study population.

Compared to low-grade subtype lung adenocarcinoma, patients with high-grade subtype lung adenocarcinoma were more likely to be younger (*p* < 0.001), had abnormal preoperative serum CEA level (*p* = 0.070), had lymphovascular invasion (*p* = 0.007), had poor differentiation (*p* = 0.001), had positive spread through air spaces (*p* = 0.001), had larger tumor size (*p* < 0.001), had more lymph node metastasis (*p* = 0.047), and had higher tumor–node–metastasis (TNM) stage (*p* = 0.030).

### 3.2. Correlations between Histological Subtypes and Clinical Outcomes

Tumor recurrence was noted in 13 patients, including 1 and 12 patients in low-grade and high-grade subtype groups, respectively.

**Figure 2:**
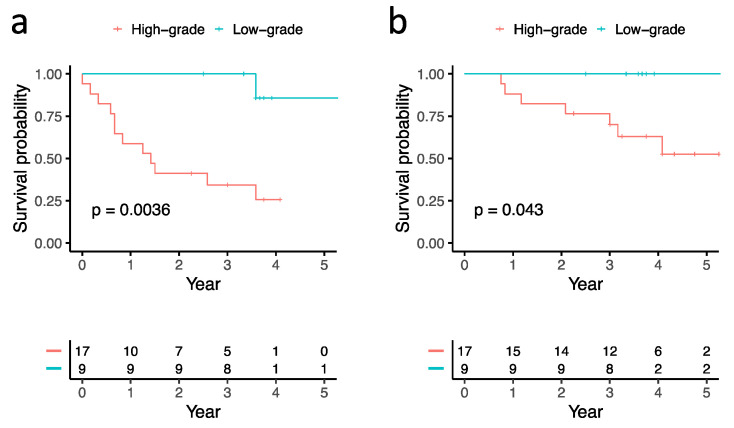
Kaplan–Meier survival analysis showed that the low-grade subtype group had superior (**a**) disease-free survival and (**b**) overall survival than the high-grade subtype group (*p* = 0.0036 and *p* = 0.043, respectively).

The 5-year DFS of the 9 patients with low-grade subtype and 17 patients with high-grade subtype were 85.7% and 25.7%, respectively (*p* = 0.0036). The 5-year OS of the 9 patients with low-grade subtype and 17 patients with high-grade subtype were 100% and 52.6%, respectively (*p* = 0.043) (Table 1, Figure 2).

### 3.3. Differentially Expressed Genes in Low-Grade (Lepidic-Predominant) and High-Grade (Solid or Micropapillary-Predominant) Adenocarcinomas

**Figure 3:**
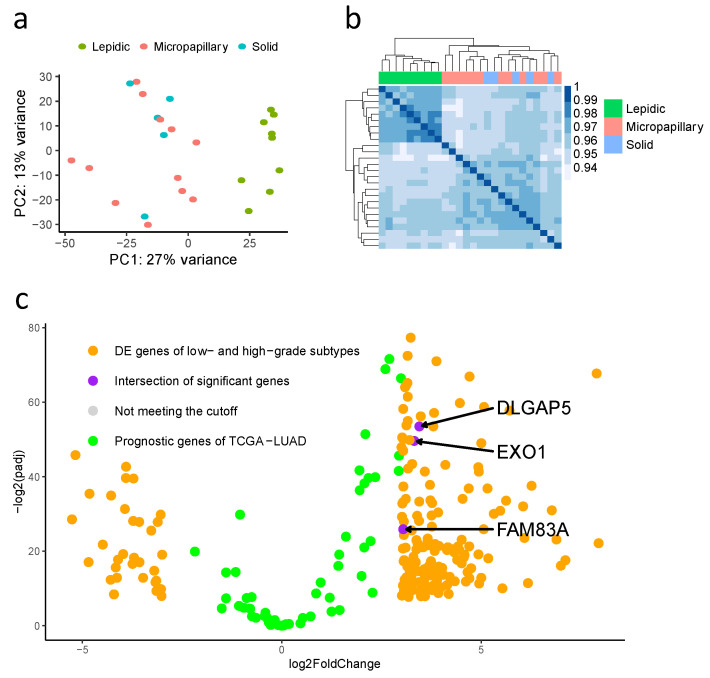
Differentially expressed genes. (**a**) Principal component analysis and (**b**) pairwise correlation analysis identified two distinct clusters associated with morphological subtypes. (**c**) Intersection of significant differentially expressed genes and The Cancer Genome Atlas-Lung Adenocarcinoma prognostic genes.

To study the difference in gene expression between high-grade and low-grade adenocarcinomas, we performed total RNA sequencing analysis. Two unsupervised methods, principal component analysis and pairwise correlation analysis, showed strong concordance in high-grade and low-grade samples. Differential gene expression analysis was applied under the criteria of adjusted *p*-value less than 0.005 and the log2 fold change being greater than and equal to 3 or less than and equal to −3. We identified 196 significant candidate genes, including 164 upregulated in high-grade adenocarcinoma and 32 upregulated in low-grade adenocarcinoma (Figure 3).

### 3.4. Construction and Validation of Three Prognostic Genes

**Figure 4:**
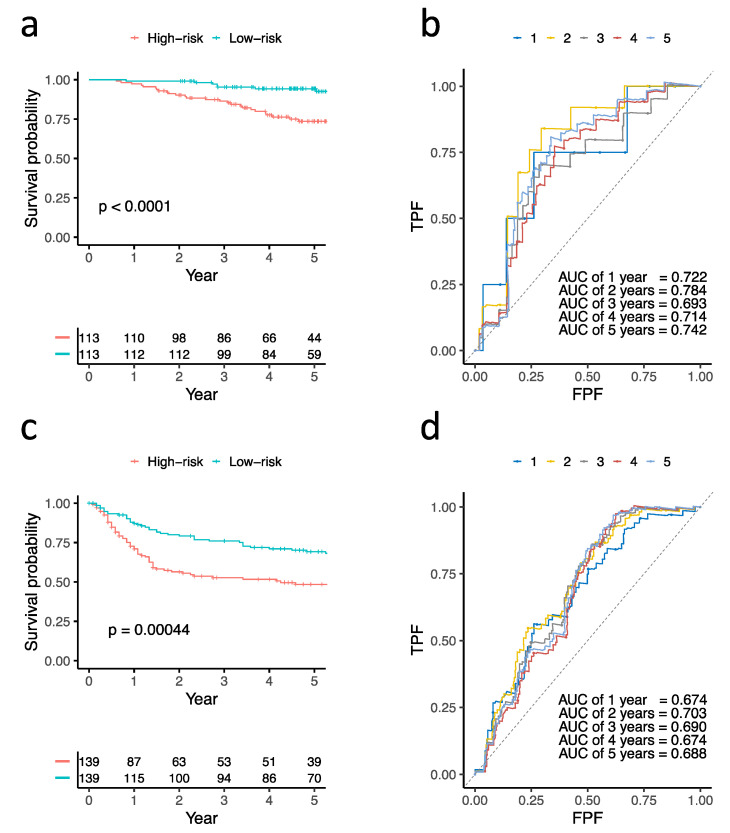
Validation of three prognostic genes by Kaplan–Meier survival analysis and time-dependent receiver operating characteristic analysis in GSE31210 (**a**,**b**) and GSE30219 (**c**,**d**). TPF: true positive fraction, FPF: false positive fraction.

The differentially expressed genes were intersected with genes fitted in a multivariate Cox proportional hazards regression model from TCGA-LUAD under the cutoff of adjusted *p*-value less than 0.005. Exonuclease 1 (EXO1), family with sequence similarity 83, member A (FAM83A), and disks large-associated protein 5 (DLGAP5) were selected as the prognostic gene signatures. A three-gene prognosis risk score was calculated based on the gene expression level multiplied by the regression coefficient. Two validation cohorts, 226 patients with adenocarcinomas of pathological stage I–II (GSE31210) and 278 patients with adenocarcinomas and squamous carcinomas (GSE30219), were used to evaluate the power of the prognostic risk score. The 226 patients with early-stage adenocarcinomas and 278 patients with adenocarcinomas and squamous carcinomas were divided into a high-risk group and a low-risk group according to their three-gene risk scores, respectively. The survival analysis showed that the high-risk group had worse OS than the low-risk group (*p*-value < 0.0001 in GSE31210 and 0.00044 in GSE30219), and the areas under the time-dependent ROC were 0.784 in GSE31210 and 0.703 in GSE30219 (Figure 4).

## 4. Discussion

Gene signatures for prognostic prediction based on gene expression profiles of patients with lung cancer have been reported in several previous studies. Dratz et al. reported a 14-gene signature for prognostic prediction in patients with non-squamous, non-small cell lung cancer (NSCLC) [24,25,26]. The reported gene signature may identify patients at high risk of mortality despite small, node-negative lung tumors and is helpful in prognostic prediction [25] and adjuvant chemotherapy selection [26] for early-stage NSCLC. Jablons et al. also reported a 15-gene signature that can differentiate between low-risk and high-risk subgroups regarding OS in patients with adenocarcinoma and squamous cell carcinoma [27]. These different gene signatures contribute to prognostic prediction and treatment decisions in patients with lung cancer. However, additional gene signatures are needed for a more accurate prognosis of lung cancer because of the diversity of prediction results. This is the first study to use a prognostic signature for lung adenocarcinoma identified by differences in gene expression profiles of low- and high-grade histological subtypes.

In this study, different gene expression profiles in low-grade (lepidic-predominant) and high-grade (solid or micropapillary-predominant) adenocarcinomas were identified. Three genes, EXO1, FAM83A, and DLGAP5 were selected as the prognostic gene signatures because they had the most differing levels of expression between the low-grade and high-grade groups after intersection with TCGA-LUAD prognostic genes. Gene expression levels of these three genes were significantly different between the high-grade and low-grade groups. In two external independent validation cohorts, the clinical outcomes of surgically resected lung cancers can be significantly predicted using this three-gene prognostic signature. Our study provided molecular evidence supporting the current pattern-based classification and grading system for lung adenocarcinoma.

EXO1 is a 5′-to-3′ exonuclease associated with DNA mismatch repair (MMR), DNA double-strand break repair, nucleotide excision repair, and immunoglobulin maturation [28,29,30,31,32]. It interacts with MSH2 and MLH in human cells and is essential for meiosis in yeasts and mice [28,32,33]. EXO1 K589E polymorphism is associated with the development of lung cancer in Taiwan and China [34,35]. High expression of EXO1 has been reported to be associated with poor prognosis in lung, prostate, and breast cancers [28,36,37]. In this study, EXO1 was significantly upregulated in lung adenocarcinomas with high-grade patterns. Since EXO1 functions as a DNA repair gene, its high expression may reflect the more complex genetic changes in the high-grade group. In this study, FAM83A was significantly upregulated in histologically high-grade adenocarcinomas compared with lepidic-predominant adenocarcinomas.

FAM83A is involved in several cell signaling pathways, including the EGFR, RAS/RAF/MEK/ERK, and PI3K/AKT/mTOR pathways [38,39,40,41,42]. Overexpression of FAM83A has been observed in lung, breast, bladder, head and neck, and cervical cancers [38,39,40,41,42]. It is highly expressed in lung adenocarcinoma, especially in those with EGFR mutations, and is considered to be a biomarker for prognosis [38,39,43]. FAM83A is associated with high proliferative activities and invasiveness of lung cancer cell lines, advanced TNM stage, and poor prognosis in patients with lung cancer [38,43]. FAM83A has been shown to promote epithelial–mesenchymal transition and Wnt signaling activation in lung adenocarcinomas, head and neck squamous cell carcinomas, and cervical squamous cell carcinomas [38,41,42,43].

The DLGAP5 gene encodes DLGAP-5, also known as hepatocarcinoma-upregulated protein (HURP). HURP is a kinetochore protein that is important in mitosis and controls spindle dynamics [44,45,46]. It is a microtubule-associated protein expressed during the cell cycle, peaking at the G2/M phase [47]. HURP was first found to be overexpressed in hepatocellular carcinoma [48]. The overexpression of HURP in cancers suggests increased mitotic rates or dysregulation of the normal cell cycle. Increased DLGAP5 gene expression has been found in most types of cancers and is associated with poor prognosis [49,50]. In lung cancer, DLGAP5 overexpression was found to correlate with decreased OS and relapse-free survival [49,50]. Our study demonstrated that DLGAP5 expression was significantly different between low- and high-grade histologic types of lung adenocarcinoma. However, the detailed mechanism by which DLGAP5 leads to poor clinical prognosis in lung cancer remains unknown.

This study has some limitations and biases. First, this was a single-center study that included only a small part of our lung cancer cohort. Further multicenter studies with larger patient populations are required. We aimed to eliminate the histological heterogeneity of analyzed tumors to select the representative genetic profiles of low- and high-grade subtypes; however, not all cases displayed 100% purity of subtype (>70% in this study). This study enrolled only patients with low-grade and high-grade histological subtypes. The performance of prognostic prediction using genetic profiles from low- and high-grade histological subtypes in general mixed-type populations needs to be further validated. The study cohort was exclusively Asian, and extrapolation to other NSCLC populations should be performed with caution. Our results showed the feasibility of using this three-gene signature for prognostic prediction in two external validation cohorts with non-Asian patients with NSCLC. Further studies using this three-gene prognostic signature are warranted in the future.

## 5. Conclusions

Using RNA sequencing, we demonstrated that histologically low- and high-grade adenocarcinomas had different gene expression profiles. We also identified prognostically related three-green signatures and validated them using two public datasets. Our study provided molecular evidence supporting the current pattern-based tumor classification and grading system for lung adenocarcinoma, as proposed by the WHO classification.

## Figures and Tables

**Table 1:** Clinicopathological characteristics and clinical outcomes.

Variables	All (*n* = 26)	Low-Grade Subtype (*n* = 9)	High-Grade Subtype (*n* = 17)	*p*-Value
age, years; mean (range)	64.7 (41–85)	67.8 (55–85)	62.9 (41–85)	<0.001
sex (female), *n* (%)	14 (53.8%)	5 (55.6%)	9 (52.9%)	0.899
smoker, *n* (%)	7 (26.9%)	2 (22.2%)	5 (29.4%)	0.694
lung cancer family history, *n* (%)	6 (23.1%)	2 (22.2%)	4 (23.5%)	0.940
abnormal serum CEA level ^a^	5 (19.2%)	0 (0%)	5 (29.4%)	0.070
visceral pleural invasion, *n* (%)	6 (23.1%)	1 (11.1%)	5 (29.4%)	0.669
lymphovascular invasion, *n* (%)	9 (34.6%)	0 (0%)	9 (52.9%)	0.007
differentiation				0.001
well/moderate	13 (50%)	8 (88.9%)	5 (29.4%)	
poor	12 (46.2%)	0 (0%)	12 (70.6%)	
STAS positive, *n* (%)	11 (42.3%)	0 (0%)	11 (64.7%)	0.001
predominant subtype, *n* (%)				<0.001
lepidic	9 (34.6%)	9 (100%)	0 (0%)	
micropapillary	12 (46.2%)	0 (0%)	12 (70.6%)	
solid	5 (19.2%)	0 (0%)	5 (29.4%)	
tumor size (cm)	2.6 ± 1.2	1.9 ± 0.6	3.0 ± 1.2	<0.001
pN stage ^b^				0.047
N0	18 (69.2%)	9 (100%)	9 (52.9%)	
N1	2 (7.7%)	0 (0%)	2 (11.8%)	
N2	6 (23.1%)	0 (0%)	6 (35.3%)	
TNM stage ^b^, *n* (%)				0.030
IA	12 (46.1%)	8 (88.9%)	4 (23.5%)	
IB	5 (19.2%)	1 (11.1%)	4 (23.5%)	
IIA	2 (7.7%)	0 (0%)	2 (11.8%)	
IIB	1 (3.8%)	0 (0%)	1 (5.9%)	
IIIA	6 (23.1%)	0 (0%)	6 (35.3%)	
surgical method				0.056
lobectomy	18 (69.2%)	4 (44.4%)	14 (82.4%)	
segmentectomy	3 (11.5%)	1 (11.1%)	2 (11.8%)	
wedge resection	5 (19.2%)	4 (44.4%)	1 (5.9%)	
clinical outcomes				
follow-up period (months)	44.2 (9–117)	51.6 (30–117)	40.9 (9–79)	
tumor recurrence	13 (50.0%)	1 (11.1%)	12 (70.6%)	
5-y DFS (%)		85.7%	25.7%	0.004
death	8 (30.8%)	1 (11.1%)	7 (41.2%)	
5-y OS (%)		100%	52.6%	0.043

CEA, carcinoembryonic antigen; DFS, disease-free survival; OS, overall survival; SD, standard deviation; STAS, spread through air spaces. ^a^ Preoperative serum CEA level of more than 5 ng/mL is defined as abnormal serum CEA level. ^b^ Tumor-node-metastasis classification for non-small lung cancer stage is based on the eighth edition of the Union for International Cancer Control and American Joint Committee on Cancer TNM classification for lung cancer.

## Data Availability

The data presented in this study are available on request from the corresponding author.
